# miR‐329‐3p in Bone Marrow Mesenchymal Stem Cells‐Derived Exosomes Promotes Bone Regeneration in Diabetic Fractures by Alleviating the SIRT3‐Mediated Oxidative Stress via Inhibiting LSD1

**DOI:** 10.1155/sci/6280416

**Published:** 2026-07-12

**Authors:** Teng Ma, Mochi Yang, Xiaoli Zhou, Xiaorui Hu, Daihao Wei, Weiwei Guo

**Affiliations:** ^1^ Department of Traumatic Orthopedics, General Hospital of Ningxia Medical University, Yinchuan, Ningxia Hui Autonomous Region, China, nxmu.edu.cn; ^2^ Department of Ophthalmology, General Hospital of Ningxia Medical University, Yinchuan, Ningxia, China, nxmu.edu.cn; ^3^ Department of Endocrinology, Cardiovascular and Cerebrovascular Disease Hospital, General Hospital of Ningxia Medical University, Yinchuan, Ningxia, China, nxmu.edu.cn; ^4^ Department of Burn, Plastic Surgery and Aesthetic Surgery, General Hospital of Ningxia Medical University, Yinchuan, Ningxia, China, nxmu.edu.cn

**Keywords:** bone regeneration, diabetic fractures, LSD1/SIRT3, miR-329-3p, the BMSCs-derived Exo

## Abstract

**Purpose:**

Influence of bone marrow mesenchymal stem cells (BMSCs)‐derived exosomes (Exo) on fracture healing in diabetes mellitus (DM) was investigated.

**Methods:**

Serum LSD1, SIRT3, superoxide dismutase (SOD), and malondialdehyde (MDA) were detected in patients with diabetic fractures. Under high‐glucose (HG) conditions, influences of LSD1, SIRT3, BMSCs‐derived Exo, and BMSCs‐derived Exo harboring miR‐329‐3p on osteogenic differentiation and oxidative stress of MC3T3‐E1 cells were assayed via ALP staining, western blotting, dichlorofluorescin diacetate (DCFH‐DA) staining, and enzyme‐linked immunosorbent assay (ELISA). Mice with diabetic fractures were treated by BMSCs, BMSCs‐derived Exo, or a combination of BMSCs and GW4869. Western blotting and immunohistochemistry examined protein expression in fracture tissues.

**Results:**

DM patients with nonunion showed higher serum LSD1 and MDA and lower serum SIRT3 and SOD than those with normal fracture healing. In HG‐induced MC3T3‐E1 cells, LSD1 silencing upregulated SIRT3, RUNX2, OPG, and SOD, increased mature osteoblasts, and reduced reactive oxygen species (ROS) and MDA; SIRT3 silencing revered these results. LSD1 downregulated SIRT3, RUNX2, OPG, and SOD, reduced mature osteoblasts, and enhanced ROS and MDA in HG‐induced MC3T3‐E1 cells; BMSCs‐derived Exo abrogated these influences. BMSCs‐derived Exo harboring miR‐329‐3p suppressed LSD1 and ROS, upregulated SIRT3, RUNX2, and OPG, and increased mature osteoblasts in HG‐induced MC3T3‐E1 cells. BMSCs transplantation in diabetic fracture mice elevated SIRT3, ALP, RUNX2, OPG, and miR‐329‐3p and reduced LSD1 in fracture tissues; miR‐329‐3p silencing in BMSCs or GW4869 treatment of mice counteracted these effects.

**Conclusion:**

BMSCs‐derived Exo harboring miR‐329‐3p induces bone regeneration in diabetic fractures by relieving the SIRT3‐mediated oxidative stress via inhibiting LSD1. It has potential in treating diabetic fractures.

## 1. Introduction

Diabetes mellitus (DM), including type I DM and type II DM, is closely related to skeletal fragility, and thus, fracture incidence is greatly increased in patients with DM [1, 2]. As a chronic disease characterized by high blood glucose, DM not only damages bone tissues but also severely impedes bone healing after fractures [3]. However, the mechanisms of poor diabetic fracture healing are still not fully understood, and there are still no effective treatments to promote diabetic fracture healing. Studies have found that a variety of diabetic complications, such as diabetic nephropathy and diabetic retinopathy, are associated with oxidative stress damage triggered by high blood glucose [4, 5]. In fact, the enhanced oxidative stress is a key factor contributing to poor fracture healing in diabetic fractures, and the oxidative stress‐induced excessive bone resorption leads to impaired fracture healing [6, 7]. Therefore, reducing oxidative stress may be an effective way to promote fracture healing in diabetic fractures.

During fracture healing, bone marrow mesenchymal stem cells (BMSCs) are key cells that promote bone regeneration [8]. BMSCs can secrete a variety of factors that modulate the microenvironment (known as the paracrine effect), and among these paracrine substances, exosomes (Exo) are considered the most valuable therapeutic factors [9]. Exo released from BMSCs are rich in bioactive components, which can exert bioactivities similar to those of BMSCs by transmitting information to damaged tissues [10]. It has been found that upregulation of miR‐19b in BMSCs can increase the level of miR‐19b in Exo; after Exo were internalized by osteoblasts, miR‐19b promotes fracture healing by targeting WW domain‐containing E3 ubiquitin protein ligase 1 (WWP1) through the Kruppel‐like factor 5 (KLF5)/β‐catenin signaling pathway [11]. miR‐136‐5p in the BMSCs‐derived Exo can facilitate fracture healing in mice by inhibiting lipoprotein‐related protein 4 (LRP4) and activating the Wnt/β‐catenin signal [12]. A recent work has highlighted that the BMSCs‐derived Exo can increase bone mass and induce bone regeneration in diabetic mice [13]. Therefore, investigating the effects of BMSCs‐derived Exo on osteogenic differentiation capacity in a high‐glucose (HG) environment will help develop effective therapeutic strategies for bone healing after diabetic fracture.

Sirtuin 3 (SIRT3) is an NAD(+)‐dependent deacetylase [14]. In DM, SIRT3 activation has been revealed to prevent diabetic nephropathy by alleviating oxidative stress [15]. Conversely, SIRT3 deficiency exacerbates poor skin wound healing via intensifying oxidative stress [16]. A recent study has emphasized the association between SIRT3 and bone regeneration in DM; SIRT3 protein is abnormally downregulated during diabetic fracture healing, leading to oxidative stress to impede fracture healing; SIRT3 agonist has shown the ability to promote fracture healing in diabetic fractures [7]. Histone lysine‐specific demethylase 1 (LSD1) is a flavin‐containing amino oxidase. Knockdown of LSD1 can enhance SIRT3 expression both in vivo and in vitro, thereby inhibiting the oxidative stress‐induced renal fibrosis; oppositely, LSD1 overexpression facilitates renal fibrosis via blocking SIRT3 expression [17]. Based on these previous findings, this article speculated that LSD1 might impede fracture healing after diabetic fractures by enhancing oxidative stress via reducing SIRT3 expression.

Given the great potential of the BMSCs‐derived Exo in treating diabetic fractures, the present work predicted miRNAs that could regulate LSD1 expression by Starbase and Targetscan online prediction software. Combined with the dual‐luciferase reporter gene assay, miR‐329‐3p was identified to regulate LSD1 expression by binding to it. As previously reported, miR‐329‐3p expression in the HG‐induced endothelial cells is reduced, while miR‐329‐3p overexpression can alleviate endothelial cell injury under the HG condition [18]. Accordingly, this work verified whether miR‐329‐3p in the BMSCs‐derived Exo could facilitate bone healing in diabetic fractures by alleviating oxidative stress via regulating LSD1/SIRT3. The current study will provide an effective strategy for the treatment of diabetic fractures.

## 2. Materials and Methods

### 2.1. Clinical Peripheral Blood Samples

Clinical peripheral blood samples were harvested from 30 healthy volunteers, 30 DM patients with normal fracture healing, and 30 DM patients with nonunion. These blood samples were centrifuged (300 × *g*, 5 min, 4°C) to collect the serum samples. Serum levels of LSD1 and SIRT3 were tested by real‐time quantitative reverse transcription‐polymerase chain reaction (qRT‐PCR) and western blotting. The serum‐derived Exo was extracted from these peripheral blood samples using an Exosomes Extraction Kit For serum (Solarbio, Beijing, China). The expression of miRNAs in the serum‐derived Exo was assayed by qRT‐PCR.

All participants have signed a written informed consent. The study was ratified by the Ethics Committee of General Hospital of Ningxia Medical University (Number KYLL‐2022‐0714) and complied with the Declaration of Helsinki.

### 2.2. Culture of Mouse Osteoblasts

Mouse osteoblasts (MC3T3‐E1 cell line) were commercially supplied by Zeye Biotechnology (Shanghai, China). Cells were grown in α‐modified minimum essential medium (α‐MEM) with 10% fetal bovine serum (FBS) at 37°C and 5% CO_2_. α‐MEM and FBS were supplied by Gibco (Thermo Fisher Scientific, Waltham, MA, USA).

For HG induction, a final dose of 30 mmol/L D‐glucose was used for treating MC3T3‐E1 cells. Specifically, an additional 24.5 mmol/L D‐glucose was added to the α‐MEM to culture MC3T3‐E1 cells since α‐MEM already contained 5.5 mmol/L D‐glucose.

### 2.3. Isolation and Culture of BMSCs

Animal experiments were approved by the Animal Ethics Committee. BLAB/c mice (8–12 weeks old) were commercially provided by KaiXue Biotechnology (Shanghai, China). Isolation of mouse BMSCs was implemented as previously reported [19]. Specifically, mice were deeply anesthetized with 5% isoflurane, followed by being euthanized. The femur and tibia were collected. Bone marrow tissues in the cavities were harvested and centrifuged to collect BMSCs. BMSCs were grown in α‐MEM with 10% FBS at 37°C and 5% CO_2_. After 8 h, BMSCs attached to the bottom of the culture flask continued to be cultured. The medium was refreshed at a 3‐day interval. After 3 weeks of culture, the purified BMSCs were obtained.

For the HG induction of BMSCs, α‐MEM with 10% FBS and a final dose of 30 mmol/L D‐glucose was used to treat BMSCs at 37°C and 5% CO_2_.

### 2.4. Oil Red O Staining and Alizarin Red Staining of BMSCs

Oil Red O staining was used to examine the lipogenic differentiation capacity of BMSCs. BMSCs were plated into 6‐well plates (1 × 10^5^ cells/mL per well) and cultivated in 1 mL of lipogenic differentiation induction medium for 14 days. The lipogenic differentiation induction medium consisted of α‐MEM with 10% FBS, 100 nM dexamethasone, 200 µM indomycin, 10 µM insulin, and 500 µM 3‐isobutyl‐1‐methylxanthine. Regarding osteogenic differentiation induction, BMSCs were maintained in 1 mL osteogenic differentiation induction medium for 14 days. The osteogenic differentiation induction medium contained α‐MEM with 10% FBS, 10 mM β‐glycerophosphate, 10 mM dexamethasone, and 50 mg/L ascorbic acid. The medium was refreshed at a 3‐day interval. After 14 days, 4% polyformaldehyde (Absin Biotechnology, Shanghai, China) was added into each well to fix BMSCs for 15 min. Then, Oil Red O staining solution (Kanglang Biotechnology, Shanghai, China) and 2% alizarin red staining solution (Kanglang Biotechnology, Shanghai, China) were separately employed to stain BMSCs for 30 min. BMSCs staining was visualized under a light microscope (Olympus, Tokyo, Japan).

### 2.5. Cell Transfection

LSD1 siRNA, SIRT3 siRNA, siRNA negative control (NC), LSD1 overexpression vectors, NC vectors, miR‐329‐3p mimics, and mimics NC were all purchased from GeneChem (Shanghai, China). By using Lipofectamine 3000 (Thermo Fisher Scientific, San Jose, CA, USA), MC3T3‐E1 cells in serum‐free α‐MEM were individually transfected by these siRNAs, vectors, or mimics. Cotransfection of MC3T3‐E1 cells was performed by using LSD1 siRNA and SIRT3 siRNA. BMSCs in serum‐free α‐MEM were transfected by miR‐329‐3p mimics, mimics NC, miR‐329‐3p inhibitors, and inhibitors NC, respectively. miR‐329‐3p inhibitors and inhibitors NC were provided by GeneChem (Shanghai, China). Following transfection, cells were fostered in α‐MEM with 10% FBS at 37°C and 5% CO_2_.

### 2.6. Isolation, Observation, and Identification of the BMSCs‐Derived Exo

BMSCs were cultivated in Exo‐free medium for 48 h at 37°C and 5% CO_2_. The culture medium of BMSCs was gathered and centrifuged (300 × *g* for 10 min and 2000 × *g* for 10 min). The supernatant was filtered by 0.22 μm sterile membrane (Biolab Technology, Beijing, China) and then centrifuged (100,000 × *g* for 2 h). The precipitation was resuspended into 15 mL of phosphate buffer saline (PBS) and centrifuged (4000 × *g*) in a centrifuge filtration device to a final volume of 200 μL. The whole procedure was implemented at 4°C. The obtained Exo was preserved under −80°C. The concentration and purity of the BMSCs‐derived Exo were 2.46 × 1010 particles/mL and 1 × 10^9^ particles per microgram of protein, respectively.

The morphology of Exo was observed under transmission electron microscopy (TEM). Exo were fixed in 1% osmium tetroxide for 2 h, dehydrated by gradient ethanol and acetone, and then loaded onto copper grids. Post stained by uranium acetate and lead citron citrate, Exo on the copper grids were placed under TEM (Hitachi H7500 TEM, Tokyo, Japan) for observation.

The size distribution of Exo was analyzed by the nanoparticle tracking analysis. The identification of Exo was carried out by detecting Exo positive markers (CD9, CD63, TSG101, HSP70, and Alix proteins) and negative markers (calnexin, GM130, and histone proteins) via using western blotting.

### 2.7. Internalization of Exo

Mouse CD63 gene was cloned into the lentiviral vector LV5, which carried a GFP sequence, to construct the LV5‐CD63 plasmid. Using Lipofectamine 3000, the LV5‐CD63 plasmid combined with lentiviral vectors was cotransfected into 293T cells. After 48 h, the lentivirus was harvested from the culture supernatant, centrifuged at 20,000 rpm for 2 h at 4°C, and resuspended in phosphate‐buffered saline. The lentivirus was serially diluted and then adapted to infect BMSCs. Post 48 h, the Exo derived from these BMSCs were gathered, followed by incubation with MC3T3‐E1 cells for 24 h. The 4′, 6‐diamidino‐2‐phenylindole (DAPI) (Kanglang Biotechnology, Shanghai, China) was used to treat MC3T3‐E1 cells for 5 min under a dark condition. The internalization of Exo by MC3T3‐E1 cells was monitored under a confocal microscope (Olympus, Tokyo, Japan).

### 2.8. Treatment and Grouping of BMSCs and Mouse Osteoblasts

For BMSCs, the following groups were set: the control group (BMSCs were cultured in normal medium); the HG group (BMSCs were maintained in HG medium); the NC group (BMSCs were transfected by mimic NC and then grown in normal medium); the mimic group (BMSCs were transfected by miR‐329‐3p mimic and then kept in normal medium); the inhibitor‐NC group (BMSCs were transfected by inhibitor NC and then cultivated in normal medium); the Inhibitor group (BMSCs were transfected with miR‐329‐3p inhibitor and then fostered in normal medium). BMSCs of each group were cultivated for 48 h at 37°C, 5% CO_2_, and then BMSCs and the BMSCs‐derived Exo were collected.

MC3T3‐E1 cells in different groups were treated as follows: the control group (MC3T3‐E1 cells were cultured in normal medium); the si‐NC group (MC3T3‐E1 cells were transfected by siRNA NC, and then cultured in normal medium); the si‐LSD1 group (MC3T3‐E1 cells were transfected by LSD1 siRNA, and then grown in normal medium); the si‐SIRT3 group (MC3T3‐E1 cells were transfected by SIRT3 siRNA, and then maintained in normal medium); the HG group (MC3T3‐E1 cells were grown in HG medium); the HG + si‐LSD1 group (MC3T3‐E1 cells were transfected by LSD1 siRNA, and then cultured in HG medium); the HG + si‐SIRT3 group (MC3T3‐E1 cells were transfected by SIRT3 siRNA, and then cultivated in HG medium); the HG + si‐LSD1 + si‐SIRT3 group (MC3T3‐E1 cells were cotransfected by LSD1 siRNA and SIRT3 siRNA, and then maintained in HG medium); the NC group (MC3T3‐E1 cells were transfected by NC vectors, and then cultured in normal medium); the LSD1 group (MC3T3‐E1 cells were transfected by LSD1 overexpression vectors, and then fosteredin normal medium); the HG + LSD1 group (MC3T3‐E1 cells were transfected by LSD1 overexpression vectors, and then kept in HG medium); the HG + Exo group (MC3T3‐E1 cells were fostered in HG medium containing 50 μL of the BMSCs‐derived Exo); the HG + LSD1 + Exo group (MC3T3‐E1 cells were transfected by LSD1 overexpression vectors, and then cultured in HG medium with 50 μL of the BMSCs‐derived Exo); the mimic group (MC3T3‐E1 cells were transfected by miR‐329‐3p mimic, and then cultured in normal medium); the HG + mimic‐Exo group (MC3T3‐E1 cells were maintained in HG medium with 50 μL of the miR‐329‐3p mimic‐transfected BMSCs‐derived Exo); the HG + inhibitor‐Exo group (MC3T3‐E1 cells were cultivated in HG medium suspended with 50 μL of the miR‐329‐3p inhibitor‐transfected BMSCs‐derived Exo). MC3T3‐E1 cells of these groups were maintained under the relevant conditions for 48 h at 37°C and 5% CO_2_.

### 2.9. Cell Counting Kit‐8 (CCK‐8) Assay

MC3T3‐E1 cells were plated in 96‐well plates (5 × 10^3^ cells per well) and cultivated in the respective conditions for 48 h at 37°C, 5% CO_2_. CCK‐8 solution (Kanglang Biotechnology, Shanghai, China) was dispersed into each well for 2 h incubation at 37°C. Absorbance values of wells were monitored under a microplate reader (Biotek, Winooski, VT, USA).

### 2.10. ALP Staining

MC3T3‐E1 cells in 6‐well plates (1 × 10^5^ cells per well) were incubated under the relevant treatment conditions for 48 h at 37°C and 5% CO_2_. ALP solution (Yubo Biotechnology, Shanghai, China) was dispersed into each well to treat MC3T3‐E1 cells for 20 min. The ALP staining observed under a light microscope (Olympus, Tokyo, Japan) was employed to evaluate mature osteoblasts.

### 2.11. Dichlorofluorescin Diacetate (DCFH‐DA) Staining

After being fostered for 48 h under the relevant conditions, MC3T3‐E1 cells in 6‐well plates were incubated with DCFH‐DA solution (20 μM) (Kanglang Biotechnology, Shanghai, China) for 45 min at 37 °C. DAPI solution was dispersed for nuclear staining. The intensity of DCFH‐DA staining was observed under a confocal microscope (Olympus, Tokyo, Japan), which was adopted to examine the reactive oxygen species (ROS) content.

### 2.12. Enzyme‐Linked Immunosorbent Assay (ELISA)

Clinical peripheral blood samples were centrifuged (300 × *g*, 5 min, 4°C), and then, serum samples were harvested. The culture medium of MC3T3‐E1 cells from different treatment groups was gathered and centrifuged (1000 × *g*, 10 min, 4°C) to collect the supernatant. Serum superoxide dismutase (SOD) and malondialdehyde (MDA) were monitored using a human SOD ELISA kit (Xinyu Biotechnology, Shanghai, China) and a human MDA ELISA kit (Xinyu Biotechnology, Shanghai, China), respectively. The SOD and MDA levels in the culture medium supernatants of MC3T3‐E1 cells were separately detected using a Mouse SOD ELISA kit (SenBeiJia Biotechnology, Nanjing, China) and a Mouse MDA ELISA kit (Biolab Technology, Beijing, China).

### 2.13. Bioinformatics Analysis and Dual Luciferase Reporter Gene Assay

Starbase combined with Targetscan online prediction software was adopted to predict miRNAs that could regulate LSD1 expression in patients with diabetic fractures. Common miRNAs were obtained by taking the intersection.

Based on the binding sites between miR‐329‐3p and LSD1 predicted by Targetscan, sequences of the wild‐type (WT)‐LSD1 and the mutant‐type (MUT)‐LSD1 were designed and synthesized by GeneChem (Shanghai, China). These sequences were loaded into luciferase reporters. MC3T3‐E1 cells in serum‐free medium were cotransfected by miR‐329‐3p mimic and luciferase reporters loaded with the WT‐LSD1 sequence, by miR‐329‐3p mimic and luciferase reporters loaded with the MUT‐LSD1 sequence, by mimic NC and luciferase reporters loaded with the WT‐LSD1 sequence, or by mimic NC and luciferase reporters loaded with the MUT‐LSD1 sequence. After transfection, MC3T3‐E1 cells were fostered in α‐MEM with 10% FBS for 48 h at 37°C and 5% CO_2_. The activity of the luciferase reporters was examined by adopting a Dual Luciferase Reporter Assay Kit (Promega, USA). Renilla luciferase activity served as the reference of Firefly luciferase activity.

### 2.14. In Vivo Study

BLAB/c mice (12 weeks old, *n* = 30) were commercially provided by KaiXue Biotechnology (Shanghai, China). Mice were housed in a specific pathogen‐free room (at 22°C, 12‐h light/dark cycle) with free access to water and diet. Animal research was approved by the Animal Ethics Committee of General Hospital of Ningxia Medical University (Number KYLL‐2022‐0714).

Construction of the mouse model of diabetic fractures was performed as previously reported [19]. First, DM induction of mice was performed by intraperitoneal injection of streptozotocin (STZ; 7.86 mL/g, once a day for 5 days). STZ was purchased from Biolab Technology (Biejing, China). One week after the last STZ injection, mice with a blood glucose concentration higher than 270 mg/dL were considered successful for DM modeling. These mice were used for the follow‐up study. Second, femoral fractures were performed on these mice with DM, strictly following the steps previously reported [20].

The 30 mice were randomly divided into five groups, and the treatment of mice in each group was carried out as follows: the model group (*n* = 6; mice only experienced the construction of the model of diabetic fractures); the model + BMSCs group (*n* = 6; after the construction of the model of diabetic fractures, mice were injected with BMSCs [2 × 10^6^ cells in 50 µL PBS [19]] in the fracture region); the model + inhibitor‐BMSCs group (*n* = 6; after the construction of the model of diabetic fractures, mice were injected with the miR‐329‐3p inhibitor transfected‐BMSCs [2 × 10^6^ cells in 50 µL PBS] in the fracture region); the model + BMSCs + GW4869 group (*n* = 6; after the construction of the model of diabetic fractures, mice were preinjected with Exo inhibitor GW4869 [2.5 μg/g; intraperitoneal injection [21]] for 1 h, and then injected with BMSCs [2 × 10^6^ cells in 50 µL PBS] in the fracture region); the control group (*n* = 6; mice were only subjected to femoral fractures). GW4869 was provided by Beyotime Biotechnology (Shanghai, China). At 22 days after the femoral fractures, mice were euthanized. The femurs of all mice were collected and preserved at −80°C.

### 2.15. qRT‐PCR

Total RNA in clinical serum, the clinical serum‐derived Exo, MC3T3‐E1 cells, BMSCs, and the BMSCs‐derived Exo were extracted using the TRIzol reagent (Absin Biotechnology, Shanghai, China). Fractured tissues of mice were ground to powder and then treated by the TRIzol reagent to isolate total RNA. Reverse transcription reaction was executed on total RNA samples with a reverse transcription kit (Lianmai Biotechnology, Shanghai, China). Then, synthesized cDNA was obtained, which was then subjected to qRT‐PCR on ABI 7500 Real‐time PCR system (Applied Biosystems, Foster City, CA, USA). The reaction procedures were 95°C for 60 s; then 95°C for 30 s, 62°C for 60 s, and 72°C for 30 s, cycled 40 times; and finally 72°C for 90s. Primers were synthesized by GeneChem (Shanghai, China). The relative expression of genes was qualified by the 2^−ΔΔCt^ method. U6 and glyceraldehyde‐3‐phosphate dehydrogenase (GAPDH) were separately regarded as the reference for miRNAs and coding genes.

### 2.16. Western Blotting

Total proteins in clinical serum, MC3T3‐E1 cells, the BMSCs‐derived Exo, and the fractured tissues of mice (ground to powder) were extracted by radio‐immuno precipitation assay lysis buffer (Kanglang Biotechnology, Shanghai, China). Post centrifugation (12,000 × *g*, 15 min, 4°C), the supernatant was subjected to the determination of the total protein concentration by employing a BCA assay kit (Kanglang Biotechnology, Shanghai, China). The total protein samples with 20 µg/lane were separated by 10% sodium dodecyl sulfate‐polyacrylamide gel electrophoresis. After being transferred to PVDF membranes (Biolab Technology, Beijing, China), the proteins were blocked in 5% skimmed milk (at room temperature, 1 h). Primary antibodies were dropped onto the PVDF membranes to react with proteins (12 h, 4°C), including mouse anti‐LSD1 (1:1000, PTM‐5938, Jingjie Biotechnology, Hangzhou, China), rabbit anti‐SIRT3 (1:1000, HY‐P86513, MCE, New Jersey, USA), rabbit anti‐RUNX2 (1:500, HY‐P80316, MCE, New Jersey, USA), mouse anti‐OPG (1:1000, HY‐P81123, MCE, New Jersey, USA), rabbit anti‐ALP (1:5000, HY‐P87188, MCE, New Jersey, USA), rabbit anti‐CD9 (1:2000, HY‐P86394, MCE, New Jersey, USA), rabbit anti‐CD63 (1:1000, HY‐P80604, MCE, New Jersey, USA), rabbit anti‐TSG101 (1:1000, HY‐P86564, MCE, New Jersey, USA), rabbit anti‐calnexin (1:1000, HY‐P80578, MCE, New Jersey, USA), rabbit anti‐HSP70 (1:500, HSP70, MCE, New Jersey, USA), rabbit anti‐Alix (1:1000, HY‐P86637, MCE, New Jersey, USA), rabbit anti‐GM130 (1:2000, HY‐P86426, MCE, New Jersey, USA), rabbit anti‐histone (1:5000, HY‐P87872, MCE, New Jersey, USA), and rabbit anti‐GAPDH (1:10000, HY‐P80137, MCE, New Jersey, USA). Thereafter, horseradish peroxidase‐conjugated goat anti‐rabbit secondary antibody (1:10000, 111‐035‐003, AmyJet Scientific, Wuhan, China) and goat anti‐mouse secondary antibody (1:20000, PTM‐7168, Jingjie Biotechnology, Hangzhou, China) were employed to treat the proteins (at room temperature, 2 h). The development of protein blots was executed by treating the PVDF membranes with the enhanced chemiluminescence reagent (Kanglang Biotechnology, Shanghai, China). The qualification of protein blots was analyzed via Image J software (ImageJ, NIH, Bethesda, MD, USA).

### 2.17. Immunohistochemistry

The fractured tissues of mice were treated with 4% paraformaldehyde for 24 h, followed by being decalcified for 21 days in 10% ethylenediaminetetraacetic acid (Solarbio, Beijing, China). The fractured tissues were encapsulated in paraffin and prepared into sections (4 µm thick). The sections were spread onto slides and then dewaxed and rehydrated using xylene and gradient alcohol, respectively. After being soaked in 3% H_2_O_2_ for 10 min, the sections were boiled for 3 min in citrate buffer. Normal goat serum (Solarbio, Beijing, China) was dropped onto the sections for 30 min blockage at 37°C. The sections were subjected to treatment with primary antibodies (12 h, 4°C), including mouse anti‐LSD1 (1:100, PTM‐5938, Jingjie Biotechnology, Hangzhou, China), rabbit anti‐SIRT3 (1:200, HY‐P86513, MCE, New Jersey, USA), rabbit anti‐RUNX2 (1:100, HY‐P80316, MCE, New Jersey, USA), mouse anti‐OPG (1:100, HY‐P81123, MCE, New Jersey, USA), and rabbit anti‐ALP (1:100, CAU32039, AmyJet Scientific, Wuhan, China). Then, the proteins were exposed to goat anti‐rabbit secondary antibody (1:500, 111‐035‐003, AmyJet Scientific, Wuhan, China) and goat anti mouse secondary antibody (1:100, PTM‐7168, Jingjie Biotechnology, Hangzhou, China) for 30 min at 37°C. Color reaction of the sections was executed by treatment with 3,3^′^‐ diaminobenzidine (Solarbio, Beijing, China). After counterstaining for 1 min with hematoxylin solution (Kanglang Biotechnology, Shanghai, China), the sections were sequentially treated with gradient alcohol and xylene. At last, the dried sections were enclosed in neutral resin and placed under a light microscope (Olympus, Tokyo, Japan) to monitor the expression of proteins.

### 2.18. Statistical Analysis

Data were displayed as mean ± standard deviation and analyzed by GraphPad Prism 6 (GraphPad Software, San Diego, CA, USA) software. A two‐tailed paired Student’s *t*‐test was adopted for data comparison between two different groups. One‐way analysis of variance combined with post hoc Tukey’s test was used for data comparison in at least three different groups. *p* < 0.05 was the threshold for a statistically significant difference.

## 3. Results

### 3.1. Poor Fracture Healing in Patients With DM Was Associated With Enhanced Oxidative Stress, Elevated LSD1, and Reduced SIRT3

**Figure 1:**
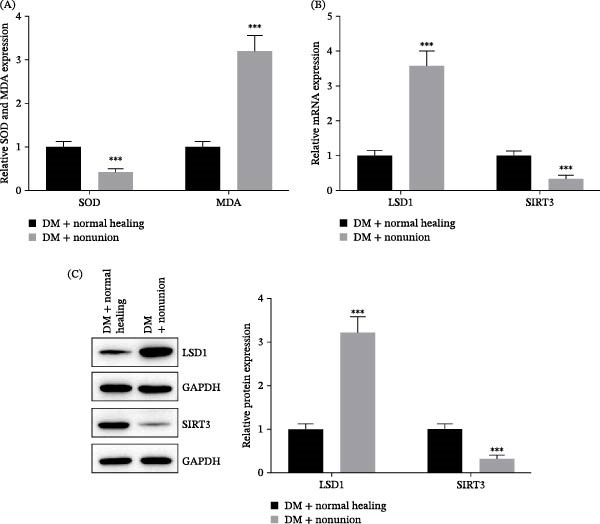
Enhanced oxidative stress, elevated LSD1, and reduced SIRT3 in serum was found in DM cases with poor fracture healing. (A) ELISA showed the decreased SOD but increased MDA in serum of DM cases with nonunion. (B and C) qRT‐PCR and western blotting presented the elevated LSD1 and reduced SIRT3 in serum of DM cases with nonunion.  ^∗∗∗^ *p* < 0.001 vs. DM cases with normal fracture healing.

Peripheral blood samples were harvested from 30 DM cases with normal fracture healing and 30 DM cases with nonunion. Oxidative stress‐related factors (including SOD and MDA) in serum were assayed by ELISA (Figure 1A), and serum levels of LSD1 and SIRT3 were scrutinized by qRT‐PCR (Figure 1B) and western blotting (Figure 1C). In comparison to DM cases with normal fracture healing, DM cases with nonunion showed lower SOD level, higher MDA level, higher LSD1 mRNA and protein expression, and lower SIRT3 mRNA and protein expression in serum (*p* < 0.001). This suggested that poor fracture healing in patients with DM might be associated with enhanced oxidative stress, elevated LSD1, and reduced SIRT3.

### 3.2. LSD1 Silencing Might Enhance the Viability of Mouse Osteoblasts in HG Environment by Upregulating SIRT3

**Figure 2:**
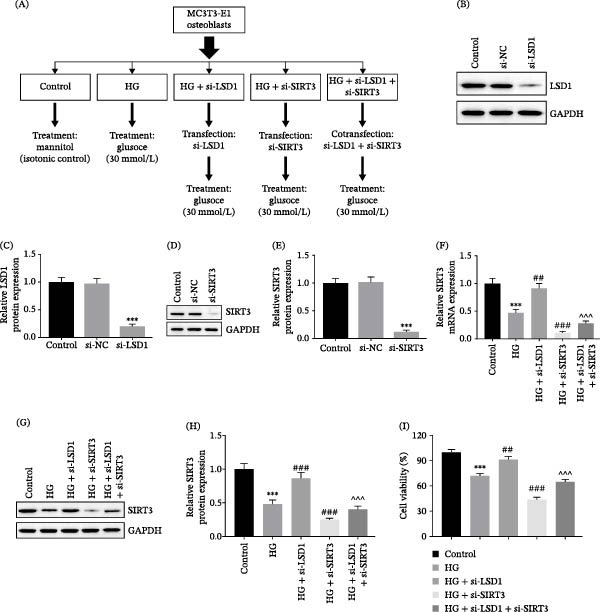
LSD1 silencing enhanced the viability of mouse osteoblasts by elevating SIRT3. (A) The flowchart detailing the procedures for MC3T3‐E1 cells in each group was prepared. (B–E) LSD1 siRNA and SIRT3 siRNA transfection effectively reduced the expression of LSD1 and SIRT3 proteins in MC3T3‐E1 cells. (F–H) LSD1 silencing elevated SIRT3 mRNA and protein expression in the HG‐induced MC3T3‐E1 cells, which was reversed by SIRT3 silencing. (I) By CCK‐8 assay, SIRT3 silencing abolished the enhancement of LSD1 silencing on the viability of the HG‐induced MC3T3‐E1 cells.  ^∗∗∗^ *p* < 0.001 vs. the control group. ##*p* < 0.01 and ###*p* < 0.001 vs. the HG group. ^^^*p* < 0.001 vs. the HG + si‐LSD1 group.

To illustrate the treatment procedures for MC3T3‐E1 cells in each group more clearly, a flowchart detailing the procedures for each group was produced (Figure 2A). For the silencing of LSD1 and SIRT3, siRNAs against LSD1 and SIRT3 were transfected into mouse osteoblasts MC3T3‐E1, respectively. By western blotting, LSD1 and SIRT3 proteins were distinctly downregulated in MC3T3‐E1 cells after transfection (*p* < 0.001) (Figure 2B–E).

MC3T3‐E1 cells were transfected with LSD1 siRNA, SIRT3 siRNA, or both. As shown by qRT‐PCR (Figure 2F) and western blotting (Figure 2G, H), HG environment caused a decrease in SIRT3 mRNA and protein in MC3T3‐E1 cells (the HG group vs. the control group) (*p* < 0.001). In HG environment, LSD1 silencing increased SIRT3 mRNA and protein in MC3T3‐E1 cells (the HG + si‐LSD1 group vs. the HG group) (*p* < 0.01, *p* < 0.001), but SIRT3 silencing reduced it (the HG + si‐SIRT3 group vs. the HG group) (*p* < 0.001). The promotion of LSD1 silencing on the expression of SIRT3 mRNA and protein in the HG‐induced MC3T3‐E1 cells was counteracted by SIRT3 silencing (the HG + si‐LSD1 + si‐SIRT3 group vs. the HG + si‐LSD1 group) (*p* < 0.001).

CCK‐8 assay (Figure 2I) was executed on MC3T3‐E1 cells to monitor the viability. HG induction of MC3T3‐E1 cells led to the attenuated viability (the HG group vs. the control group) (*p* < 0.001). LSD1 silencing enhanced viability in the HG‐induced MC3T3‐E1 cells (the HG + si‐LSD1 group vs. the HG group) (*p* < 0.01, *p* < 0.001). However, SIRT3 silencing showed an opposite influence (the HG + si‐SIRT3 group vs. the HG group) (*p* < 0.001). As referred to the HG + si‐LSD1 group, MC3T3‐E1 cells of the HG + si‐LSD1 + si‐SIRT3 group displayed lower viability (*p* < 0.001). Thus, LSD1 silencing enhanced the viability of MC3T3‐E1 cells in the HG environment but was abrogated by SIRT3 silencing. This suggested that LSD1 silencing might intensify the viability of MC3T3‐E1 cells in the HG environment by increasing SIRT3.

### 3.3. LSD1 Silencing Might Facilitate Osteogenic Differentiation and Block Oxidative Stress of Mouse Osteoblasts in HG Environment by Enhancing SIRT3 Expression

**Figure 3:**
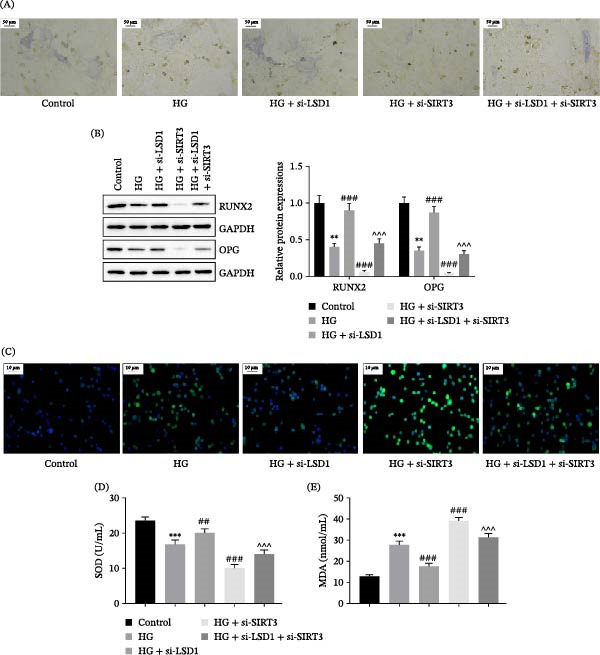
LSD1 silencing facilitate osteogenic differentiation and relieved oxidative stress of mouse osteoblasts in HG environment by increasing SIRT3. (A) ALP staining indicated that SIRT3 silencing counteracted the promotion of LSD1 silencing on mature osteoblasts in the HG‐induced MC3T3‐E1 cells. (B) Western blotting implied that SIRT3 silencing reversed the promotion of LSD1 silencing on the expression of RUNX2 and OPG proteins in the HG‐induced MC3T3‐E1 cells. (C) DCFH‐DA staining showed that SIRT3 silencing reversed the suppression of LSD1 silencing ROS production in the HG‐induced MC3T3‐E1 cells. (D and E) ELISA implied that the promotion of LSD1 silencing on SOD, and its suppression on MDA, in the culture medium of the HG‐induced MC3T3‐E1 cells was abrogated by SIRT3 silencing.  ^∗∗^ *p* < 0.01 and  ^∗∗∗^ *p* < 0.001 vs. the control group. ##*p* < 0.01 and ###*p* < 0.001 vs. the HG group. ^^^*p* < 0.001 vs. the HG + si‐LSD1 group.

ALP staining (Figure 3A) and western blotting (Figure 3B) were performed on MC3T3‐E1 cells to explore the mature osteoblasts and osteogenic differentiation markers, separately. HG exposure caused the reduced mature osteoblasts and expression of RUNX2 and OPG proteins (the HG group vs. the control group) (*p* < 0.001). LSD1 silencing increased mature osteoblasts and expression of RUNX2 and OPG proteins in the HG‐induced MC3T3‐E1 cells (the HG + si‐LSD1 group vs. the HG group) (*p* < 0.01, *p* < 0.001). SIRT3 silencing displayed an opposite impact (the HG + si‐SIRT3 group vs. the HG group) (*p* < 0.001). In contrast to the HG + si‐LSD1 group, MC3T3‐E1 cells of the HG + si‐LSD1 + si‐SIRT3 group had the reduced mature osteoblasts and expression of RUNX2 and OPG proteins (*p* < 0.001). Thereby, SIRT3 silencing reversed the enhancement of LSD1 silencing on the osteogenic differentiation of MC3T3‐E1 cells in the HG environment. This was indicative that LSD1 silencing might facilitate osteogenic differentiation of MC3T3‐E1 cells in HG environment by increasing SIRT3.

To examine the influence of LSD1 and SIRT3 on oxidative stress, DCFH‐DA staining (Figure 3C) and ELISA (Figure 3D, E) were conducted. MC3T3‐E1 cells in HG environment showed the intensified ROS staining, lower SOD level, but higher level of MDA (the HG group vs. the control group) (*p* < 0.001). LSD1 silencing of the HG‐induced MC3T3‐E1 cells displayed the attenuated ROS staining, increased SOD level and decreased MDA level, comparatively (the HG + si‐LSD1 group vs. the HG group) (*p* < 0.01, *p* < 0.001). Oppositely, SIRT3 silencing enhanced ROS staining, reduced SOD level, and elevated MDA level in the HG‐induced MC3T3‐E1 cells (the HG + si‐SIRT3 group vs. the HG group) (*p* < 0.001). When matched to MC3T3‐E1 cells of the HG + si‐LSD1 group, those of the HG + si‐LSD1 + si‐SIRT3 group exhibited an exacerbation of ROS staining, reduction of SOD level, but elevation of MDA level (*p* < 0.001). Hence, LSD1 silencing alleviated oxidative stress of MC3T3‐E1 cells in the HG environment, which was counteracted by SIRT3 silencing. LSD1 silencing was thus suggested to relieve oxidative stress of MC3T3‐E1 cells in HG environment by increasing SIRT3.

### 3.4. Isolation and Characterization of BMSCs and BMSCs‐Derived Exo

**Figure 4:**
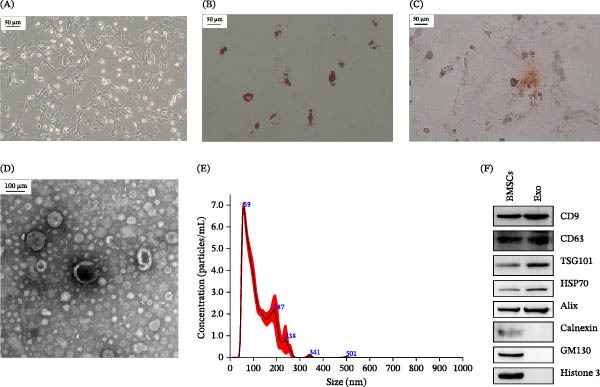
Isolation and characterization of BMSCs and BMSCs‐derived Exo. (A) BMSCs isolated from mouse bone marrow were cultured. (B) Oil Red O staining showed favorable lipogenic differentiation ability of BMSCs. (C) Alizarin red staining exhibited favorable osteogenic differentiation capacity of BMSCs. (D) The BMSCs‐derived Exo showed round or oval shapes under TEM. (E) Nanoparticle tracking analysis showed that the size distribution of the BMSCs‐derived Exo was mainly between 50–150 nm. (F) By western blotting, the BMSCs‐derived Exo were capable of expressing Exo‐positive markers (CD9, CD63, and TSG101 proteins), rather than negative markers (including calnexin, GM130, and histone proteins).

BMSCs were isolated from mouse bone marrow and cultured (Figure 4A). According to Oil Red O staining (Figure 4B) and alizarin red staining (Figure 4C), the isolated BMSCs had favorable capacities for lipogenic differentiation and osteogenic differentiation. The BMSCs‐derived Exo were isolated, which showed round or oval shapes under TEM (Figure 4D). By nanoparticle tracking analysis, the size distribution of the BMSCs‐derived Exo was primarily between 50–150 nm (Figure 4E). The BMSCs‐derived Exo had the ability to express Exo‐positive markers (CD9, CD63, TSG101, HSP70, and Alix proteins) but rarely expressed Exo‐negative markers (including calnexin, GM130, and histone proteins) (Figure 4F). Thereby, the BMSCs‐derived Exo were successfully isolated.

### 3.5. The BMSCs‐Derived Exo Might Enhance the Osteogenic Differentiation of Mouse Osteoblasts in HG Environment by Down‐Modulating LSD1 in Mouse Osteoblasts

**Figure 5:**
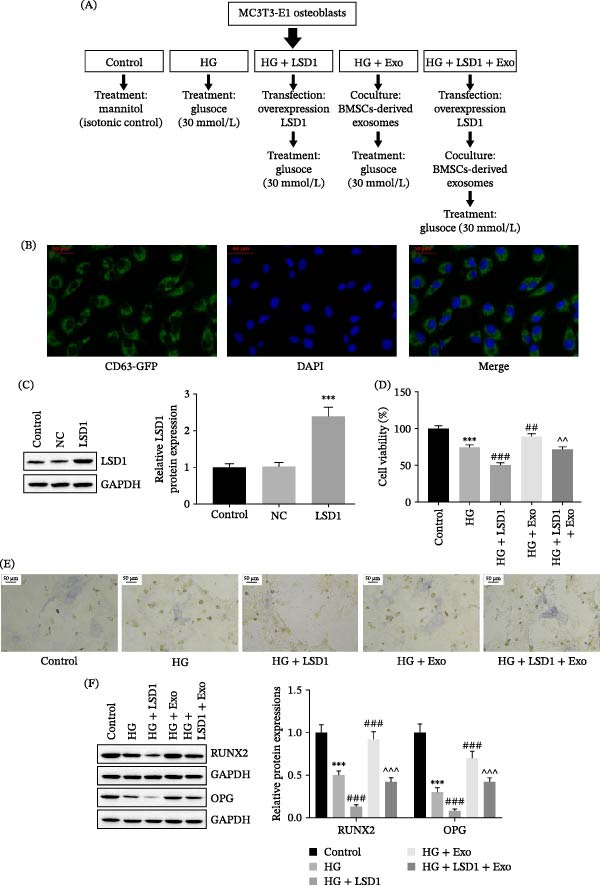
The BMSCs‐derived Exo facilitated the osteogenic differentiation of mouse osteoblasts in HG environment by reducing LSD1 in mouse osteoblasts. (A) The treatment of MC3T3‐E1 cells in each group was clearly illustrated in a flowchart. (B) Under a confocal microscope, the BMSCs‐derived Exo (acquired from the LV5‐CD63‐GFP‐infected BMSCs) could be internalized by MC3T3‐E1 cells. (C) LSD1 overexpression vectors effectively upregulated LSD1 protein in MC3T3‐E1 cells, as demonstrated by western blotting. (D) CCK‐8 presented that the BMSCs‐derived Exo treatment intensified MC3T3‐E1 cell viability, but this influence was eliminated by LSD1 overexpression in MC3T3‐E1 cells. (E) ALP staining suggested that the promotion of the BMSCs‐derived Exo treatment on mature osteoblasts was abrogated by LSD1 overexpression in MC3T3‐E1 cells. (F) From western blotting, the BMSCs‐derived Exo treatment enhanced the expression of RUNX2 and OPG proteins in MC3T3‐E1 cells, which was reversed by LSD1 overexpression in MC3T3‐E1 cells.  ^∗∗∗^ *p* < 0.001 vs. the control group. ##*p* < 0.01 and ###*p* < 0.001 vs. the HG group. ^^*p* < 0.01 and ^^^*p* < 0.001 vs. the HG + Exo group.

The treatment of MC3T3‐E1 cells in each group was clearly illustrated in Figure 5A. For the observation of uptake, the BMSCs‐derived Exo that acquired from the LV5‐CD63‐GFP‐infected BMSCs was incubated with MC3T3‐E1 cells. It could be observed that the BMSCs‐derived Exo could be taken up by MC3T3‐E1 cells (Figure 5B). To construct the LSD1‐overexpressed MC3T3‐E1 cells, LSD1 overexpression vectors were transfected into MC3T3‐E1 cells. By western blotting, LSD1 overexpression vectors significantly enhanced LSD1 expression in MC3T3‐E1 cells (the LSD1 group vs. the control group) (*p* < 0.001) (Figure 5C).

The BMSCs‐derived Exo was used to treat MC3T3‐E1 cells under HG conditions. CCK‐8 assay (Figure 5D), ALP staining (Figure 5E), and western blotting (Figure 5F) were implemented to assay MC3T3‐E1 cell viability, mature osteoblasts, and osteogenic differentiation markers, respectively. Under HG conditions, MC3T3‐E1 cells presented lower viability, less mature osteoblasts, and lower expression of RUNX2 and OPG proteins (the HG group vs. the control group) (*p* < 0.001). LSD1 overexpression further impaired MC3T3‐E1 cell viability and reduced mature osteoblasts, and expression of RUNX2 and OPG proteins under HG condition (the HG + LSD1 group vs. the HG group) (*p* < 0.01, *p* < 0.001). Interestingly, the BMSCs‐derived Exo treatment of the HG‐induced MC3T3‐E1 cells enhanced its viability, increased mature osteoblasts and expression of RUNX2 and OPG proteins (the HG + Exo group vs. the HG group) (*p* < 0.01, *p* < 0.001). However, in contrast to the HG + Exo group, MC3T3‐E1 cells of the HG + LSD1 + Exo group displayed reduced viability and decreased mature osteoblasts and expression of RUNX2 and OPG proteins (*p* < 0.01, *p* < 0.001). Thus, the BMSCs‐derived Exo treatment enhanced the osteogenic differentiation of MC3T3‐E1 cells under the HG condition, which was abolished by LSD1 overexpression in MC3T3‐E1 cells. Therefore, the BMSCs‐derived Exo might facilitate the osteogenic differentiation of MC3T3‐E1 cells under HG conditions by reducing LSD1 expression.

### 3.6. The BMSCs‐Derived Exo Might Promote SIRT3 Expression and Repress Oxidative Stress in Mouse Osteoblasts Under HG Condition by Reducing LSD1 in Mouse Osteoblasts

**Figure 6:**
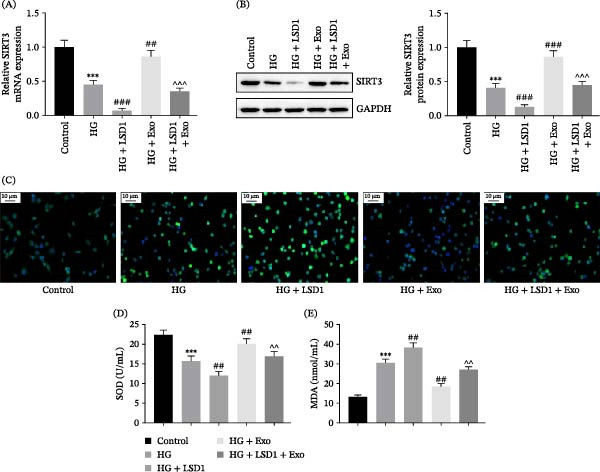
The BMSCs‐derived Exo enhanced SIRT3 and suppressed oxidative stress in the HG‐induced mouse osteoblasts by reducing LSD1. (A and B) According to qRT‐PCR and Western blotting, the BMSCs‐derived Exo treatment elevated SIRT3 mRNA and protein in MC3T3‐E1 cells under HG conditions, which was abrogated by LSD1 overexpression in MC3T3‐E1 cells. (C) DCFH‐DA staining of MC3T3‐E1 cells indicated that the BMSCs‐derived Exo treatment reduced ROS production in MC3T3‐E1 cells under HG condition, but was reversed by LSD1 overexpression in MC3T3‐E1 cells. (D and E) ELISA implied that the BMSCs‐derived Exo treatment increased SOD level and decreased MDA level in the culture medium of the HG‐induced MC3T3‐E1 cells. LSD1 overexpression in MC3T3‐E1 cells abrogated this effect.  ^∗∗∗^ *p* < 0.001 vs. the control group. ##*p* < 0.01 and ###*p* < 0.001 vs. the HG group. ^^*p* < 0.01 and ^^^*p* < 0.001 vs. the HG + Exo group.

SIRT3 in MC3T3‐E1 cells of different groups was examined through qRT‐PCR (Figure 6A) along with western blotting (Figure 6B). HG induction caused a much decrease in SIRT3 mRNA and protein in MC3T3‐E1 cells (the HG group vs. the control group) (*p* < 0.001). In HG environment, LSD1 overexpression in MC3T3‐E1 cells downregulated SIRT3 mRNA and protein (the HG + LSD1 group vs. the HG group) (*p* < 0.001). The BMSCs‐derived Exo treatment of MC3T3‐E1 cells up‐modulated it (the HG + Exo group vs. the HG group) (*p* < 0.01). In fact, the promotion of the BMSCs‐derived Exo treatment on SIRT3 mRNA and protein in the HG‐induced MC3T3‐E1 cells was eliminated by LSD1 overexpression in MC3T3‐E1 cells (the HG + LSD1 + Exo group vs. the HG + Exo group) (*p* < 0.001).

DCFH‐DA staining of MC3T3‐E1 cells (Figure 6C) and ELISA of the culture medium (Figure 6D, E) were performed to research ROS level and the levels of SOD and MDA, respectively. The HG‐induced MC3T3‐E1 cells showed the intensified ROS production, decreased SOD level, and elevated MDA level (the HG group vs. the control group) (*p* < 0.001). For the HG‐induced MC3T3‐E1 cells, LSD1 overexpression further enhanced ROS production, reduced SOD, and increased MDA (the HG + LSD1 group vs. the HG group) (*p* < 0.01). On the contrary, the BMSCs‐derived Exo treatment of the HG‐induced MC3T3‐E1 cells decreased ROS production, elevated SOD level, and diminished MDA level (the HG + Exo group vs. the HG group) (*p* < 0.01). However, these effects of the BMSCs‐derived Exo on the HG‐induced MC3T3‐E1 cells were abolished by LSD1 overexpression in MC3T3‐E1 cells (the HG + LSD1 + Exo group vs. the HG + Exo group) (*p* < 0.01). Thereby, the BMSCs‐derived Exo was implied to increase SIRT3 and block oxidative stress in the HG‐induced MC3T3‐E1 cells by reducing LSD1.

### 3.7. The Elevated LSD1 in Patients With Diabetic Fracture Was Associated With miR‐329‐3p Downregulation

**Figure 7:**
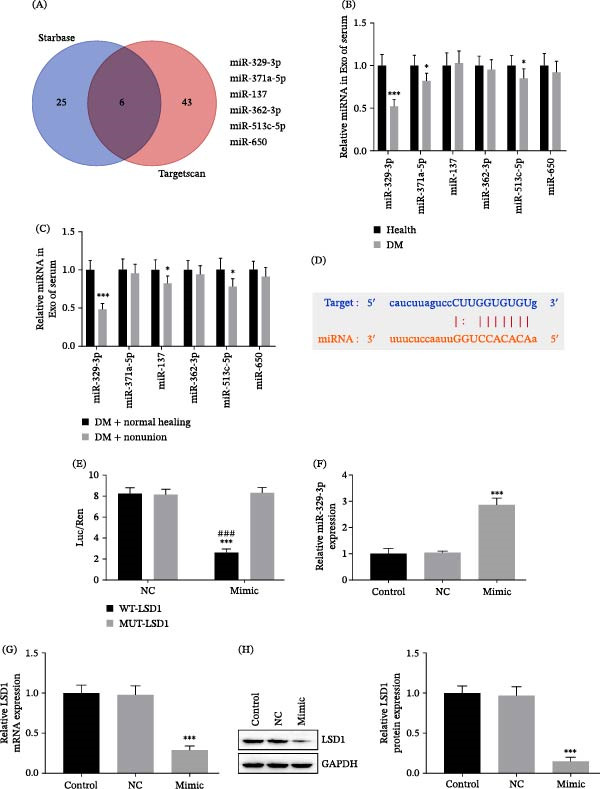
miR‐329–3 p was downregulated in patients with diabetic fracture, and it directly suppressed LSD1 expression. (A) miRNAs that could regulate LSD1 expression in patients with diabetic fractures were predicted by Starbase and Targetscan. Six common miRNAs were obtained by taking the intersection. (B) The expression of the six common miRNAs in the serum‐derived Exo of DM patients and healthy volunteers was assayed by qRT‐PCR.  ^∗^ *p* < 0.05 and  ^∗∗∗^ *p* < 0.001 vs. healthy volunteers. (C) The expression of the six common miRNAs in the serum‐derived Exo of DM cases with normal fracture healing and DM cases with nonunion was examined by qRT‐PCR.  ^∗^ *p* < 0.05 and  ^∗∗∗^ *p* < 0.001 vs. DM cases with normal fracture healing. (D) The binding site between miR‐329‐3p and LSD1 as predicted by Targetscan. (E) Dual luciferase reporter gene assay revealed the binding between miR‐329‐3p and LSD1.  ^∗∗∗^ *p* < 0.001 vs. MUT‐LSD1. ###*p* < 0.001 vs. NC. (F) By qRT‐PCR, miR‐329‐3p mimic transfection effectively upregulated miR‐329‐3p in MC3T3‐E1 cells.  ^∗∗∗^ *p* < 0.001 vs. the control group. (G and H) Based on qRT‐PCR and western blotting, miR‐329‐3p overexpression distinctly reduced the expression of LSD1 mRNA and protein in MC3T3‐E1 cells.  ^∗∗∗^ *p* < 0.001 vs. the control group.

To analyze miRNAs that could regulate LSD1 expression in patients with diabetic fractures, we employed two prediction tools (i.e., Starbase and Targetscan) to predict miRNAs that possessed bind sites for LSD1. By taking the intersection, a total of six common miRNAs were obtained, including miR‐329‐3p, miR‐371a‐5p, miR‐362‐3p, miR‐650, miR‐513c‐5p, and miR‐137 (Figure 7A). Then, by qRT‐PCR, the expression of the six miRNAs was assayed in the serum‐derived Exo of patients with DM. Relative to healthy volunteers, distinctly lower expression of miR‐329‐3p, miR‐371a‐5p, and miR‐513c‐5p was found in the serum‐derived Exo of patients with DM (*p* < 0.05, *p* < 0.001) (Figure 7B). In addition, the serum‐derived Exo was isolated from 30 DM cases with normal fracture healing and 30 DM cases with nonunion. qRT‐PCR showed a diminish in the expression of miR‐329‐3p, miR‐137, and miR‐513c‐5p in the serum‐derived Exo of the 30 DM cases with nonunion, as matched to those of the 30 DM cases with normal fracture healing (*p* < 0.05, *p* < 0.001) (Figure 7C). Thus, miR‐329‐3p was chosen for the subsequent study as the decrease in its expression was most pronounced. The binding site between miR‐329‐3p and LSD1 predicted by Targetscan is shown in Figure 7D. By dual luciferase reporter gene assay, miR‐329‐3p mimic significantly diminished the relative luciferase activity of the WT‐LSD1 reporter (*p* < 0.001), rather than MUT‐LSD1 reporter (Figure 7E). Furthermore, miR‐329‐3p mimic were transfected into MC3T3‐E1 cells to research the influence of miR‐329‐3p on LSD1. As displayed by Figure 7F, miR‐329‐3p mimic led to a much greater elevation in miR‐329‐3p (the mimic group vs. the control group) (*p* < 0.001). This indicated that MC3T3‐E1 cells were successfully transfected by the miR‐329‐3p mimic. More importantly, miR‐329‐3p mimic caused a prominent decrease in LSD1 mRNA and protein in MC3T3‐E1 cells (the mimic group vs. the control group) (*p* < 0.001) (Figure 7G, H). Thus, these results validated the inhibition of miR‐329‐3p on LSD1.

### 3.8. miR‐329‐3p in the BMSCs‐Derived Exo Promoted Osteogenic Differentiation of Mouse Osteoblasts Under HG Condition

**Figure 8:**
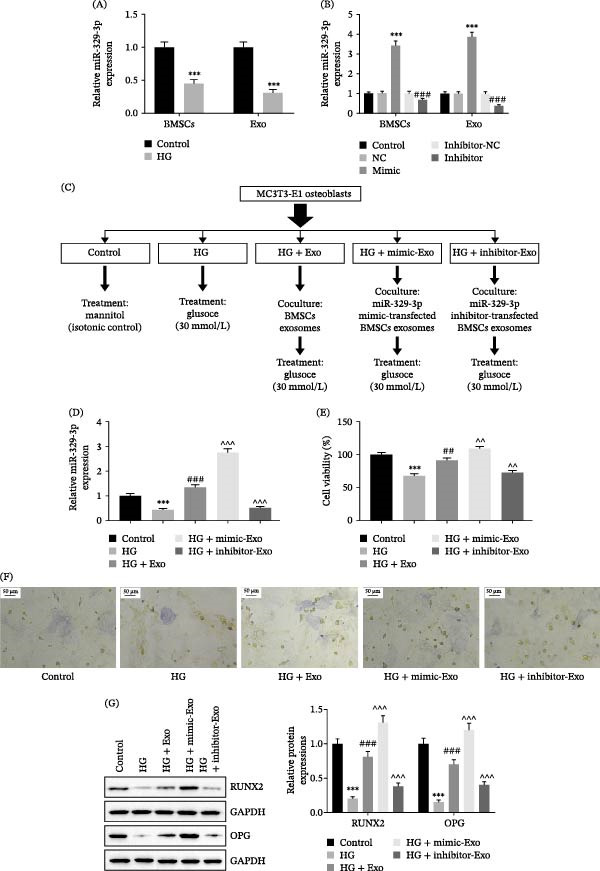
miR‐329‐3p in the BMSCs‐derived Exo facilitated osteogenic differentiation of mouse osteoblasts under HG condition. (A) The flowchart detailing the treatment procedures of MC3T3‐E1 cells in each group. (B) By qRT‐PCR, HG induction of BMSCs reduced the expression of miR‐329‐3p in BMSCs and the BMSCs‐derived Exo. (C) qRT‐PCR showed that the level of miR‐329‐3p in BMSCs and the BMSCs‐derived Exo was effectively modulated by transfecting miR‐329‐3p mimic or inhibitor into BMSCs. (D) As qRT‐PCR implied, miR‐329‐3p in the BMSCs‐derived Exo increased the expression of miR‐329‐3p in MC3T3‐E1 cells under HG condition. (E) CCK‐8 assay suggested that, miR‐329‐3p in the BMSCs‐derived Exo enhanced the viability of MC3T3‐E1 cells under HG condition. (F) ALP staining revealed that, miR‐329‐3p in the BMSCs‐derived Exo increased mature osteoblasts in MC3T3‐E1 cells under HG condition. (G) Western blotting indicated that, miR‐329‐3p in the BMSCs‐derived Exo intensified the expression of osteogenic differentiation proteins (RUNX2 and OPG) in MC3T3‐E1 cells under HG condition.  ^∗∗∗^ *p* < 0.001 vs. the control group. ##*p* < 0.01 and ###*p* < 0.001 vs. the HG group. ^^*p* < 0.01 and ^^^*p* < 0.001 vs. the HG + Exo group.

BMSCs were cultured under HG conditions, and the Exo were collected. qRT‐PCR showed that, under HG conditions, BMSCs expressed lower miR‐329‐3p (the HG group vs. the control group) (*p* < 0.001). HG induction of BMSCs caused a much diminished miR‐329‐3p in the BMSCs‐derived Exo (the HG group vs. the control group) (*p* < 0.001) (Figure 8A). Subsequently, the miR‐329‐3p mimic and its inhibitor were separately transfected into BMSCs. miR‐329‐3p mimic markedly increased miR‐329‐3p expression in BMSCs and the BMSCs‐derived Exo (the mimic group vs. the control group) (*p* < 0.001). Conversely, miR‐329‐3p inhibitor led to a significant decrease in miR‐329‐3p expression in BMSCs and the BMSCs‐derived Exo (the inhibitor group vs. the control group) (*p* < 0.001) (Figure 8B). Thus, miR‐329‐3p mimic as well as inhibitor effectively modulated the level of miR‐329‐3p in the BMSCs‐derived Exo.

Exo‐derived from BMSCs, the miR‐329‐3p mimic‐transfected BMSCs, and the miR‐329‐3p inhibitor‐transfected BMSCs were used to treat MC3T3‐E1 cells under HG conditions. The processing procedure of MC3T3‐E1 cells in each group is shown in Figure 8C. qRT‐PCR (Figure 8D), CCK‐8 assay (Figure 8E), ALP staining (Figure 8F), and western blotting (Figure 8G) were implemented on MC3T3‐E1 cells to monitor miR‐329‐3p expression, MC3T3‐E1 cell viability, mature osteoblasts, and the expression of osteogenic differentiation markers, respectively. Under HG conditions, MC3T3‐E1 cells showed lower miR‐329‐3p expression, lower cell viability, less mature osteoblasts, and lower expression of RUNX2 and OPG proteins (the HG group vs. the control group) (*p* < 0.001). The BMSCs‐derived Exo treatment upregulated miR‐329‐3p expression, enhanced cell viability, increased mature osteoblasts, and elevated RUNX2 and OPG proteins in MC3T3‐E1 cells under HG condition (the HG + Exo group vs. the HG group) (*p* < 0.01, *p* < 0.001). In contrast to MC3T3‐E1 cells treated by the BMSCs‐derived Exo (the HG + Exo group), those treated by the miR‐329‐3p mimic‐transfected BMSCs‐derived Exo (the HG + mimic‐Exo group) showed higher miR‐329‐3p expression, higher cell viability, more mature osteoblasts, and higher expression of RUNX2 and OPG proteins under HG condition (*p* < 0.01, *p* < 0.001). However, under HG condition, MC3T3‐E1 cells treated by the miR‐329‐3p inhibitor‐transfected BMSCs‐derived Exo had the reduced miR‐329‐3p expression, the attenuated cell viability, less mature osteoblasts, and the downregulated RUNX2 and OPG proteins, comparatively (the HG + inhibitor‐Exo group vs. the HG + Exo group) (*p* < 0.01, *p* < 0.001). Hence, miR‐329‐3p in the BMSCs‐derived Exo enhanced osteogenic differentiation of MC3T3‐E1 cells under the HG condition.

### 3.9. miR‐329‐3p in the BMSCs‐Derived Exo Reduced LSD1, Increased SIRT3, and Relieved Oxidative Stress in Mouse Osteoblasts Under HG Condition

**Figure 9:**
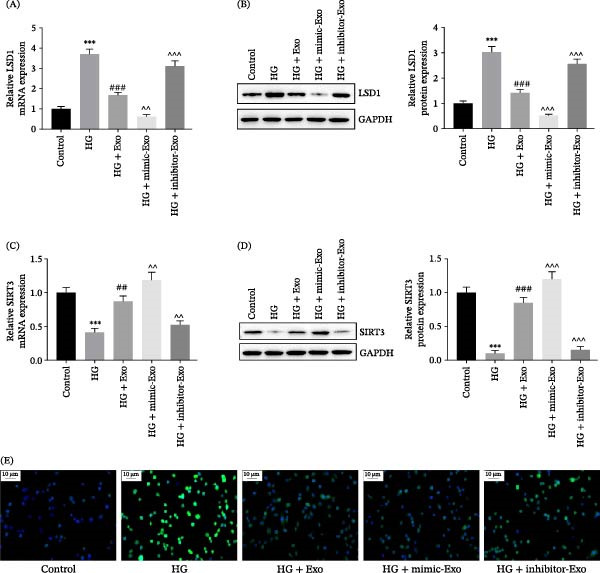
miR‐329‐3p in the BMSCs‐derived Exo diminished LSD1, elevated SIRT3, and mitigated oxidative stress in mouse osteoblasts under HG condition. (A and B) As indicated by qRT‐PCR and western blotting, miR‐329‐3p in the BMSCs‐derived Exo decreased LSD1 mRNA and protein expression in MC3T3‐E1 cells under HG condition. (C and D) miR‐329‐3p in the BMSCs‐derived Exo enhanced SIRT3 mRNA and protein expression in MC3T3‐E1 cells under HG condition, according to qRT‐PCR and western blotting. (E) DCFH‐DA staining presented that, miR‐329‐3p in the BMSCs‐derived Exo reduced ROS production in MC3T3‐E1 cells under HG condition.  ^∗∗∗^ *p* < 0.001 vs. the control group. ##*p* < 0.01 and ###*p* < 0.001 vs. the HG group. ^^*p* < 0.01 and ^^^*p* < 0.001 vs. the HG + Exo group.

LSD1 and SIRT3 expression in MC3T3‐E1 cells of different groups were appraised by qRT‐PCR and western blotting. MC3T3‐E1 cells under HG conditions expressed higher LSD1 mRNA and protein and lower SIRT3 mRNA and protein (the HG group vs. the control group) (*p* < 0.001). The BMSCs‐derived Exo treatment reduced LSD1 and increased SIRT3 comparatively (the HG + Exo group vs. the HG group) (*p* < 0.01, *p* < 0.001). When referred to the HG + Exo group, MC3T3‐E1 cells treated by the miR‐329‐3p mimic‐transfected BMSCs‐derived Exo (the HG + mimic‐Exo group) expressed lower LSD1 and higher SIRT3 (*p* < 0.01, *p* < 0.001). Compared to the HG + Exo group, MC3T3‐E1 cells exposed to the miR‐329‐3p inhibitor‐transfected BMSCs‐derived Exo (the HG + inhibitor‐Exo group) showed higher LSD1 expression and lower SIRT3 expression (*p* < 0.01, *p* < 0.001) (Figure 9A–D). Thereby, miR‐329‐3p in the BMSCs‐derived Exo suppressed LSD1 expression and increased SIRT3 expression in MC3T3‐E1 cells under the HG condition.

According to DCFH‐DA staining (Figure 9E), MC3T3‐E1 cells under HG conditions showed the increased ROS level (the HG group vs. the control group). The BMSCs‐derived Exo treatment reduced the production of ROS in MC3T3‐E1 cells under the HG condition, comparatively (the HG + Exo group vs. the HG group). In contrast to MC3T3‐E1 cells treated by the BMSCs‐derived Exo (the HG + Exo group), those treated by the miR‐329‐3p mimic‐transfected BMSCs‐derived Exo (the HG + mimic‐Exo group) displayed reduced ROS production. Oppositely, the miR‐329‐3p inhibitor‐transfected BMSCs‐derived Exo treatment caused an increase in ROS production under the HG condition (the HG + inhibitor‐Exo group vs. the HG + Exo group). Thus, miR‐329‐3p in the BMSCs‐derived Exo could attenuate oxidative stress in MC3T3‐E1 cells under the HG condition.

### 3.10. miR‐329‐3p Silencing or Exo Inhibitor GW4869 Counteracted the Promotion of BMSCs on Fracture Healing in Mice With Diabetic Fractures

**Figure 10:**
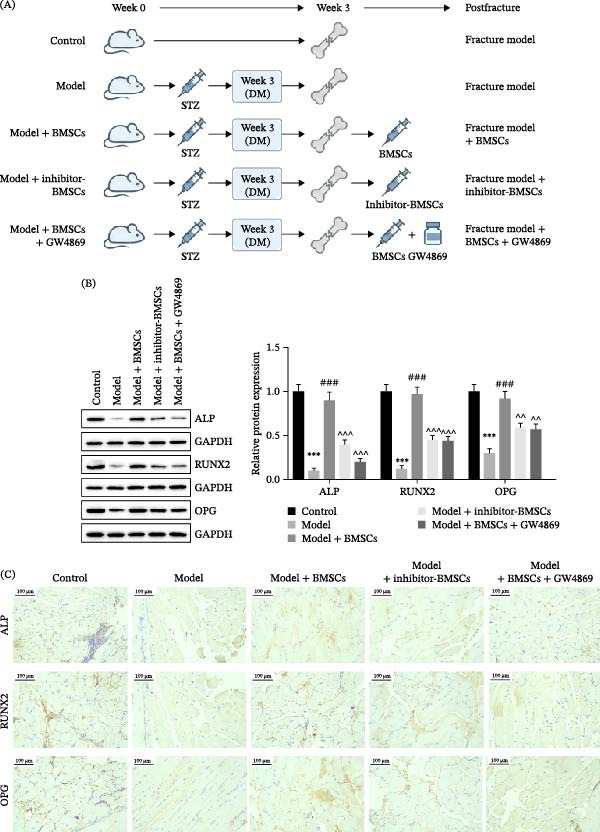
miR‐329‐3p silencing or Exo inhibitor GW4869 reversed the promotion of BMSCs on fracture healing in mice with diabetic fractures. (A) The flowchart of the animal experiments. (B and C) Based on western blotting and immunohistochemistry results, BMSCs transplantation enhanced the expression of ALP, RUNX2 and OPG proteins in fractured tissues of mice with diabetic fractures, but this influence was abrogated by miR‐329‐3p silencing or Exo inhibitor GW4869.  ^∗∗∗^ *p* < 0.001 vs. the control group. ###*p* < 0.001 vs. the model group. ^^*p* < 0.01 and ^^^*p* < 0.001 vs. the model + BMSCs group.

The mouse model of diabetic fractures was with BMSCs transplantation (the model + BMSCs group), with the miR‐329‐3p inhibitor transfected‐BMSCs transplantation (the model + inhibitor‐BMSCs group), or with BMSCs transplantation accompanied by intraperitoneal injection of Exo inhibitor GW4869 (the model + BMSCs + GW4869 group). The flowchart of the animal experiments is shown in Figure 10A. Osteogenic proteins were detected in fractured tissues by western blotting. Lower expression of ALP, RUNX2, and OPG proteins were found in fractured tissues of mice in the model group (the model group vs. the control group) (*p* < 0.001). BMSCs transplantation elevated ALP, RUNX2, and OPG proteins in fractured tissues of mice significantly (the model + BMSCs group vs. the model group) (*p* < 0.001). However, compared to the model + BMSCs group, mice of the model + inhibitor‐BMSCs group and the model + BMSCs + GW4869 group expressed lower ALP, RUNX2, and OPG proteins in fractured tissues (*p* < 0.01, *p* < 0.001) (Figure 10B). Similar expression trends of ALP, RUNX2, and OPG proteins were discovered in immunohistochemical results of fractured tissues (Figure 10C). Based on these data, it was miR‐329‐3p in the BMSCs‐derived Exo that facilitated fracture healing in mice with diabetic fractures.

### 3.11. miR‐329‐3p Silencing or Exo Inhibitor GW4869 Reversed the Regulation of BMSCs on the Expression of LSD1 and SIRT3 in Fractured Tissues of Mice With Diabetic Fractures

**Figure 11:**
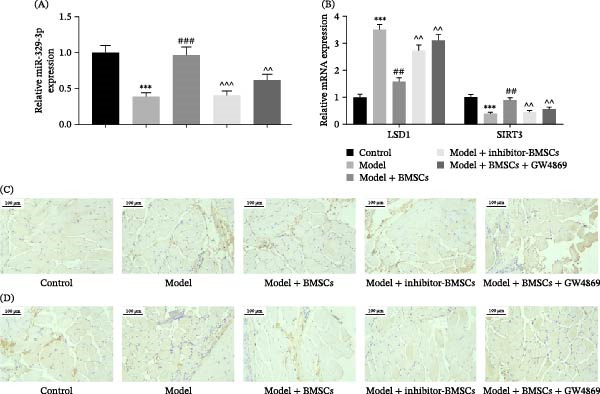
miR‐329‐3p silencing or Exo inhibitor GW4869 reversed the regulation of BMSCs on LSD1 and SIRT3 expression in fractured tissues of mice with diabetic fractures. (A) qRT‐PCR indicated that, BMSCs transplantation increased miR‐329‐3p expression in fractured tissues of mice with diabetic fractures, which was reversed by miR‐329‐3p silencing or Exo inhibitor GW4869. (B) According to qRT‐PCR, BMSCs transplantation downregulated LSD1 mRNA and upregulated SIRT3 mRNA in fractured tissues of mice with diabetic fractures. This influence was eliminated by miR‐329‐3p silencing or Exo inhibitor GW4869. (C and D) By immunohistochemistry, BMSCs transplantation attenuated LSD1 expression and enhanced SIRT3 expression in fractured tissues of mice with diabetic fractures but was abolished by miR‐329‐3p silencing or Exo inhibitor GW4869.  ^∗∗∗^ *p* < 0.001 vs. the control group. ##*p* < 0.01 and ###*p* < 0.001 vs. the model group. ^^*p* < 0.01 and ^^^*p* < 0.001 vs. the model + BMSCs group.

The expression of miR‐329‐3p in fractured tissues of mice was appraised by qRT‐PCR. Mice with diabetic fractures expressed lower miR‐329‐3p in fractured tissues (the model group vs. the control group) (*p* < 0.001). BMSCs transplantation elevated miR‐329‐3p in fractured tissues of mice with diabetic fractures (the model + BMSCs group vs. the model group) (*p* < 0.001). Conversely, miR‐329‐3p silencing or GW4869 treatment downregulated miR‐329‐3p in fractured tissues of mice with diabetic fractures (the model + inhibitor‐BMSCs group and the model + BMSCs + GW4869 group vs. the model + BMSCs group, respectively) (*p* < 0.01, *p* < 0.001) (Figure 11A).

qRT‐PCR was executed on fractured tissues to scrutinize LSD1 and SIRT3 in the fractured tissues of mice. The increased LSD1 mRNA but declined SIRT3 mRNA was monitored in fractured tissues of mice with diabetic fractures (the model group vs. the control group) (*p* < 0.001). By BMSCs transplantation, LSD1 mRNA expression was reduced and SIRT3 mRNA expression was enhanced in fractured tissues of mice with diabetic fractures (the model + BMSCs group vs. the model group) (*p* < 0.01). However, the suppression of BMSCs transplantation on LSD1 mRNA expression, as well as its promotion on SIRT3 mRNA expression, in fractured tissues of mice with diabetic fractures was counteracted by miR‐329‐3p silencing or GW4869 treatment (the model + inhibitor‐BMSCs group and the model + BMSCs + GW4869 group vs. the model + BMSCs group, respectively) (*p* < 0.01) (Figure 11B). Immunohistochemistry showed the similar trend in LSD1 expression (Figure 11C) and SIRT3 (Figure 11D) in fractured tissues of mice in different groups. Thus, miR‐329‐3p in the BMSCs‐derived Exo might facilitate fracture healing in mice with diabetic fractures by reducing LSD1 and elevating SIRT3.

## 4. Discussion

This study revealed the upregulated LSD1 and the downregulated SIRT3 in patients with diabetic fractures. The BMSCs‐derived Exo showed promotion of osteogenic differentiation and inhibition of oxidative stress in MC3T3‐E1 cells under the HG condition. Specifically, the BMSCs‐derived Exo harboring miR‐329‐3p could enhance osteogenic differentiation of HG‐induced MC3T3‐E1 cells by alleviating oxidative stress. This process might be involved in the mechanism that miR‐329‐3p in the BMSCs‐derived Exo elevated SIRT3 by repressing LSD1 in the HG‐induced MC3T3‐E1 cells. In vivo experiment indicated that miR‐329‐3p in the BMSCs‐derived Exo facilitated fracture healing in mice with diabetic fractures. These discoveries suggested the BMSCs‐derived Exo harboring miR‐329‐3p to be an effective strategy in treating diabetic fractures.

DM causes osteoporosis, increases the risk of fracture, and impedes fracture healing [22]. Under the DM condition, cellular dysfunction due to excessive oxidative stress is a critical factor in poor fracture healing [23]. Studies have shown that oxidative stress can inhibit osteoblast differentiation and lead to osteoclast damage and apoptosis [24, 25]. MDA is the major aldehyde product of lipid peroxidation during oxidative stress, and its overproduction exacerbates cellular damage [26]. As an essential metalloenzyme, SOD acts as the first line of defense against ROS. It alleviates oxidative stress by decomposing superoxide radicals and cleaving hydrogen peroxides and hydroperoxides [27]. Therefore, MDA and SOD are the most commonly used markers for assessing oxidative stress in a variety of diseases, including DM [27]. This article showed aberrantly increased serum MDA and reduced serum SOD in DM patients with nonunion relative to that in DM patients with normal fracture healing. This suggested exacerbated oxidative stress in DM patients with nonunion.

SIRT3 is an important regulator of antioxidant stress response, which can be expressed in the nucleus and mitochondria [28, 29]. It is widely involved in modulating diabetes complications, such as diabetic retinopathy, diabetes‐associated cognitive dysfunction, and diabetic nephropathy [30–32]. The exacerbated oxidative stress and impeded fracture healing have been found in rats with diabetic fractures; it may be attributed to the reduced SIRT3 expression; actually, SIRT3 activation facilitates diabetic fracture healing in rats [7]. However, more information about SIRT3 in regulating diabetic fracture healing remains unavailable. This work revealed reduced SIRT3 expression in DM patients with nonunion. Moreover, under the HG condition, SIRT3 silencing reduced RUNX2 and OPG proteins, increased ROS and MDA, and declined SOD in MC3T3‐E1 cells. RUNX2 and OPG are osteogenic markers that participate in promoting bone regeneration [33]. The diminished expression of RUNX2 and OPG has been discovered in mice with diabetic fractures [34]. ROS is an important intermediate of oxidative stress, associating with osteogenic differentiation and bone regeneration; excessive ROS accumulation leads to osteoblast apoptosis and impaired bone regeneration [24]. In DM, hyperglycemia and insulin resistance can result in mitochondrial dysfunction to induce the overproduction of ROS [35]. Thus, SIRT3 silencing was implied to block osteogenic differentiation and exacerbate oxidative stress in MC3T3‐E1 cells in HG environments.

The role of LSD1 in diabetic fracture healing has yet to be clearly revealed. LSD1 is considered a negative regulator of osteogenic differentiation, and its knockdown promotes the osteogenic differentiation of human adipose‐derived stem cells [36]. LSD1 downregulation can induce osteogenic differentiation of human periodontal ligament stem cells (hPDLSCs) in periodontitis [37]. Similarly, this article showed elevated LSD1 expression in DM patients with nonunion. Previous studies have implied that LSD1 knockdown can alleviate the DM‐induced renal fibrosis by activating SIRT3 expression [17]. According to this article, LSD1 silencing elevated SIRT3 expression, enhanced osteogenic differentiation, and attenuated oxidative stress in the HG‐induced MC3T3‐E1 cells. However, SIRT3 silencing abolished these above influences of LSD1 silencing on the HG‐induced MC3T3‐E1 cells. Hence, LSD1 silencing might induce osteogenic differentiation of MC3T3‐E1 cells under the HG condition by alleviating oxidative stress via upregulating SIRT3.

More importantly, the BMSCs‐derived Exo was suggested to be an effective strategy in treating diabetic fractures as it promoted osteogenic differentiation and relieved oxidative stress in MC3T3‐E1 cells under the HG condition. However, these effects were reversed by LSD1 overexpression in MC3T3‐E1 cells. Besides, the BMSCs‐derived Exo elevated SIRT3 expression in the HG‐induced MC3T3‐E1 cells, which was counteracted by LSD1 overexpression. This evidence suggested that the BMSCs‐derived Exo might facilitate osteogenic differentiation of the HG‐induced MC3T3‐E1 cells by attenuating the SIRT3‐mediated oxidative stress via suppressing LSD1. The BMSCs‐derived Exo are enriched with a large number of active substances; it can promote the proliferation as well as differentiation of osteoblasts by targeting signal transducer and activator of transcription 1 (STAT1) expression via delivery of miR‐935 [38]. This work identified that miR‐329‐3p could inhibit the LSD1 expression in MC3T3‐E1 cells by binding to it. Research on miR‐329‐3p in the fields of bone regeneration and osteogenic differentiation is still rarely reported. In DM‐related research, only a report indicated that miR‐329‐3p can relive the HG‐induced endothelial cell injury [18]. In the current study, the BMSCs‐derived Exo harboring miR‐329‐3p was found to reduce LSD1, elevate SIRT3, intensified osteogenic differentiation, and mitigate oxidative stress in MC3T3‐E1 cells under the HG condition. By in vivo data, BMSCs transplantation in mice with diabetic fracture promoted bone regeneration and fracture healing, reduced LSD1 expression, and increased SIRT3 expression in fractured tissues. However, these influences were counteracted by miR‐329‐3p silencing in BMSCs or GW4869 treatment of mice. Thus, all of these results were indicative that the BMSCs‐derived Exo harboring miR‐329‐3p facilitated bone regeneration in diabetic fractures by mitigating the SIRT3‐mediated oxidative stress via inhibiting LSD1. This work suggested the BMSCs‐derived Exo harboring miR‐329‐3p to be an effective strategy in treating diabetic fractures. It should be noted that, as a commonly used Exo inhibitor, GW4869 may also affect cellular pathways and signal transduction other than Exo biosynthesis, and therefore potential off‐target effects may exist. According to previous study, GW4869 can significantly inhibit the release of Exo in mice at an intraperitoneal dose of 2.5 μg/g [21]. Thus, this study selected 2.5 μg/g of GW4869 to treat the mice. Despite the potential for off‐target effects, the use of GW4869 in the in vivo experiment in this article was capable of reflecting, to a certain extent, the therapeutic role of the BMSCs‐derived Exo in fracture healing.

Research on Exo therapy for fractures has garnered widespread attention within the medical community. This article also revealed the potential of the BMSCs‐derived Exo in treating diabetic fractures. However, treatment of diabetic fractures with the BMSCs‐derived Exo still faces significant challenges in clinical translation. Issues regarding large‐scale production, batch variability, storage stability, delivery strategies, and targeting efficiency remain unresolved. These critical problems, which limit the clinical translation of the BMSCs‐derived Exo, still require urgent resolution. Furthermore, the long‐term safety, immunogenicity, and potential adverse effects of Exo‐based therapy are key considerations for clinical translation. These critical factors also limit the clinical translation of Exo. Therefore, although this article suggests the potential of the BMSCs‐derived Exo for treating diabetic fractures, the evidence is far from sufficient to support its clinical translation. Consequently, extensive in‐depth research will still be required in the future to advance the clinical application of the BMSCs‐derived Exo in the treatment of diabetic fractures.

This study has some limitations. This study established a mouse model of diabetic fractures and assessed fracture healing 22 days after injury. The 22‐day postfracture assessment primarily reflects the early repair process of the bone. This relatively short observation period may not adequately reflect long‐term outcomes such as bone remodeling, mechanical strength, and functional recovery. Extending the study duration following fracture would help to enhance its clinical relevance. However, due to laboratory limitations, this valuable research topic cannot currently be pursued, and it will be a priority for our future research.

## 5. Conclusion

The current study indicated the enhanced oxidative stress, the elevated LSD1, and the reduced SIRT3 in patients with diabetic fractures. The BMSCs‐derived Exo was effective in treating diabetic fractures since it facilitated osteogenic differentiation and suppressed oxidative stress in MC3T3‐E1 cells under the HG condition. Both in vitro and in vivo data indicated that miR‐329‐3p in the BMSCs‐derived Exo could induce osteogenic differentiation and bone regeneration in diabetic fractures by attenuating the SIRT3‐mediated oxidative stress via repressing LSD1. Thus, the BMSCs‐derived Exo harboring miR‐329‐3p is proposed to be an effective strategy in treating diabetic fractures, with promising clinical applications.

## Author Contributions

Weiwei Guo designed the study. Teng Ma and Mochi Yang performed the bioinformatic analyses. Xiaoli Zhou, Xiaorui Hu, and Daihao Wei analyzed and interpreted data. Teng Ma wrote the manuscript.

## Funding

This work was supported by the Ningxia Natural Science Foundation (Grant2023AAC03578).

## Disclosure

All authors have read, reviewed, edited, and agreed to the published version of the manuscript.

## Ethics Statement

All participants have signed a written informed consent. The study was ratified by the Ethics Committee of General Hospital of Ningxia Medical University (Number KYLL‐2022‐0714) and complied with the Declaration of Helsinki. Animal research was approved by the Animal Ethics Committee of General Hospital of Ningxia Medical University (N umber KYLL‐2022‐0714).

## Conflicts of Interest

The authors declare no conflicts of interest.

## Data Availability

All data generated or analyzed in this study are available from the corresponding author upon reasonable request.
